# A homozygous nonsense mutation identified in *COL7A1* in a family with autosomal recessive dystrophic epidermolysis bullosa

**DOI:** 10.25122/jml-2024-0090

**Published:** 2024-09

**Authors:** Muhammad Ayub, Xing Xiong, Saima Anwer, Janine Altmüller, Muhammad Naeem, Noor Hassan, Kafaitullah Khan, Susanne Motameny, Samira Khaliq, Fazal Ur Rehman, Syed Ashraf Uddin, Abdul Wali, Regina Betz, Sulman Basit

**Affiliations:** 1 Institute of Biochemistry, University of Balochistan, Quetta, Pakistan; 2 Institute of Human Genetics, Medical Faculty & University Hospital Bonn, University of Bonn, Bonn, Germany; 3 Cologne Center for Genomics, University of Cologne, Cologne, Germany; 4 Department of Microbiology, University of Balochistan, Quetta, Pakistan; 5 University of Information Technology and Management Sciences, Quetta, Pakistan; 6 Center for Genetics and Inherited Diseases, Taibah University Almadinah, Medina, Kingdom of Saudi Arabia

**Keywords:** epidermolysis bullosa, whole exome sequencing, nonsense mutation, *COL7A1* gene

## Abstract

Autosomal recessive dystrophic epidermolysis bullosa (RDEB) is a severe form of an inherited skin disorder. RDEB segregates both in an autosomal dominant as well as in an autosomal recessive pattern. It has been shown that both forms of dystrophic epidermolysis bullosa (DEB) are caused by mutations in the *COL7A1* gene. In this study, we investigated a consanguineous four-generation family with two individuals displaying the RDEB phenotype. Both patients showed multiple skin erosions, atrophic scares, crusted scaling, and pseudosyndactyly. Whole exome sequencing (WES) was performed to identify the underlying genetic defect, revealing a homozygous nonsense mutation, c.409C>T (p.Arg137*) in *COL7A1* in both patients. This variant was validated through Sanger sequencing and confirmed to segregate within the family. This report describes a recurrent nonsense mutation in *COL7A1* that leads to a severe form of autosomal recessive dystrophic epidermolysis bullosa. Moreover, this study demonstrates that whole exome sequencing analysis is imperative in resolving clinically and genetically heterogeneous diseases like RDEB. Furthermore, this study expands the mutation spectrum of the *COL7A1* gene in distinct populations.

## INTRODUCTION

Dystrophic epidermolysis bullosa (DEB) is a clinically and genetically heterogeneous disorder of the skin [1]. It is characterized by cutaneous and mucosal fragility, which results in generalized skin blisters and scar formation. Common clinical manifestations include skin blisters and erosions, scars due to blistering of internal mucosal membranes, and pseudosyndactyly with an appearance of mitten hand and foot [2]. Extensive variability has been observed in the clinical features of epidermolysis bullosa (EB), ranging from mild to severe forms. In severe cases, symptoms extend beyond the skin and can involve muscles and internal organs, including the esophagus [3]. Common manifestations in severe EB include esophageal strictures and muscular dystrophy [3]. Mutations in at least 20 genes have been identified as causes of various forms of EB. Based on the underlying genetic defect and organs involved, hereditary EB has been divided into several types and subtypes, including epidermolysis bullosa simplex (autosomal dominant), junctional epidermolysis bullosa (autosomal recessive), and dystrophic epidermolysis bullosa (autosomal dominant or recessive) [4]. The most severe form is autosomal recessive dystrophic EB (RDEB), characterized by widespread skin lesions and involvement of the basement membrane [5]. Mutations in the *COL7A1* gene are the most frequent cause of RDEB phenotype. *COL7A1* encompasses a 31 kb region and consists of 118 coding exons [6]. It encodes a long 9 kb mRNA and a 350 kDa polypeptide chain [7,8]. A Gly-X-Y repeat sequence forms a collagenous segment that folds into a triple helical confirmation and finally forms a type VII collagen molecule, the main component of the anchoring fibrils at the dermal-epidermal junction (DEJ) [9]. *COL7A1* is highly expressed in keratinocytes and dermal fibroblasts, which are the major source of anchoring fibrils [10,11]. In RDEB, deficiency of anchoring fibrils leads to an abnormal morphology and defective functioning of DEJ [12,13].

This study aimed to clinically assess a family with two children affected by autosomal recessive dystrophic epidermolysis bullosa and to identify the underlying genetic mutation through comprehensive genetic analysis.

## Material and Methods

### Subjects

**Figure 1:**
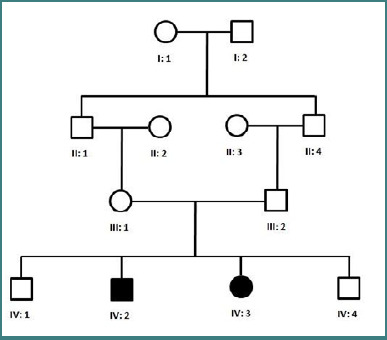
Pedigree of a family segregating autosomal recessive epidermolysis bullosa. Squares and circles represent men and women, respectively.

A consanguineous four-generation family with two individuals showing clinical features of autosomal recessive epidermolysis bullosa was investigated (Figure 1). Family members were interviewed, and a family pedigree was drawn, followed by the collection of blood samples from five individuals, including both parents (III:2, III:3), both affected individuals (IV:1, IV:2), and an unaffected sibling (IV:3). Both affected and unaffected family members were clinically examined by a consultant dermatologist at the Civil Hospital Quetta, Pakistan.

### DNA extraction

Genomic DNA was extracted using the QIAmp DNA Blood Midi Kit (QIAGEN). DNA purity was determined with a spectrophotometer and DNA visualization was performed using ethidium bromide-stained 1% agarose gel. DNA samples were stored at -20 °C.

### Whole exome sequencing

Whole exome sequencing (WES) was performed on the DNA of one affected individual (IV:1) at the Cologne Center for Genomics (CCG, University of Cologne, Germany). Briefly, 1 microgram of DNA was sonicated to generate >800 bps fragments. A single 'A' nucleotide was added to the 3' ends of the blunt-ended, phosphorylated fragments. An adaptor with a single base 'T' overhang was ligated to both ends of the genomic fragments. Ligated products were purified, and unligated products were removed. Agarose gel electrophoresis was used for size selection, and a 2 mm wide gel slice containing DNA of the desired size was excised.

**Figure 2:**
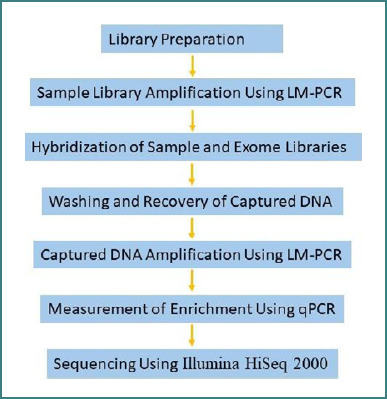
Workflow for SeqCap EZ Exome Library Experiments Using Illumina HiSeq 2000 instrument. LM-PCR, Ligation-mediated polymerase chain reaction

The adaptors containing fragments were selectively enriched by PCR amplification. The PCR was performed with two primers that annealed to the ends of the adaptors. The SeqCap EZ Human Exome Library version 2.0 kit (Roche NimbleGen) was used for the enrichment process. The resulting library was purified and size-selected by agarose gel electrophoresis and Agilent Bioanalyzer, followed by quantitation by PicoGreen. Library quantitation and quantification are very important for optimum cluster densities across every lane of the flow cell. The enriched libraries were subjected to sequencing using the paired-end protocol on an Illumina HiSeq 2000 (Illumina) instrument (Figure 2).

Exome data filtering was performed with the Varbank pipeline v.2.18 (https://varbank.ccg.uni-koeln.de/). Variants were analyzed, and genes related to skin disorders, genodermatoses, ectodermal dysplasia and especially epidermolysis bullosa were initially screened for pathogenic variants.

### Polymerase chain reaction (PCR)

Briefly, 2µl (20ng/µl) genomic DNA was subjected to amplification in a 25µl reaction mixture containing 5µl GC rich buffer, 1µl forward primer (10 pmol), 1µl reverse primer (10pmol), 0.5µl accu Prime GC rich DNA polymerase (2U/µl) and 15.5µl PCR grade deionized water. Thermal cycling conditions used for the amplification were 95 °C for 5 min followed by 95°C for 30s, 57 °C for 30s, 72°C for 1 min (35 cycles), and a final extension at 72°C for 10 mins.

### Sanger sequencing

Primers were designed for the amplification of exonic regions containing exome-discovered variants of interest. Primer 3 tool was used to pick specific primers for the region of interest. The primers used were TGGAGAATGACAGAACGAAGG and TGTGATCAGGATGCAGACC. The annealing temperature used for the amplification of exon 3 of the *COL7A1* gene was 59 °C, and the resulting product was 397 bp. Sanger sequencing of the affected individuals and their family members was conducted to verify the discovered variant using the BigDye Terminator v1.1 Cycle Sequencing kit (Applied Biosystems) and an ABI3100 genetic analyzer (Applied Biosystems). Variant location was determined using the Seqman software and comparisons with the wild-type sequence, which was extracted from the UCSC Genome Browser website (http://genome.ucsc.edu).

## RESULTS

### Clinical features of the patients

**Figure 3:**
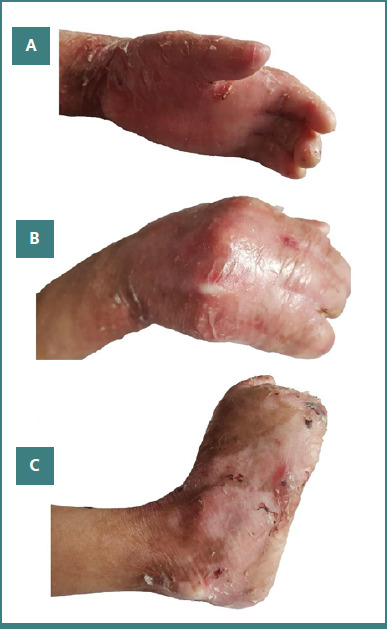
Clinical presentation of an affected individual (IV-2) showing a severe form of autosomal recessive dystrophic epidermolysis bullosa. Both patients (IV-2, IV-3) showed multiple skin erosions, atrophic scares, crusted scaling, and pseudo-syndactyly. Dental decay and loss of nails were also observed. Partial fusion of digits and webbed fingers and toes, and bleeding blisters and lesions are evident.

Both affected individuals manifested a severe form of dystrophic epidermolysis bullosa. They showed erosions and bullae formation on their whole body, accompanied by severe bleeding blisters. Clinical features were observed right after birth. A small lesional patch could be observed in the abdominal region, which spread over the whole body over time, affecting mainly hands, feet, neck, dorsal and abdominal region. The patients in this case were severely malnourished because they had problems swallowing food due to the blisters in the oral cavity (Supplementary Table 1). Teeth deformities, dental decay, and loss of nails were also observed. Physical examination detected the involvement of multiple internal organs as evident from improper functioning of digestion (gastrointestinal tract involvement), genitourinary system involvement (recurrent urinary tract infection), growth retardation, extremely weak immune system, severe anemia, partial fusion of digits and webbed fingers and toes, and pseudo-syndactyly (Figure 3). Patients complained about skin itching and recurrent blisters at the knees and elbows. Severe skin fragility and itching with slow healing of painful bleeding blisters and lesions disrupted daily routine activities.

Supplementary Table 1

### Exome data analysis and segregation analysis

**Figure 4:**
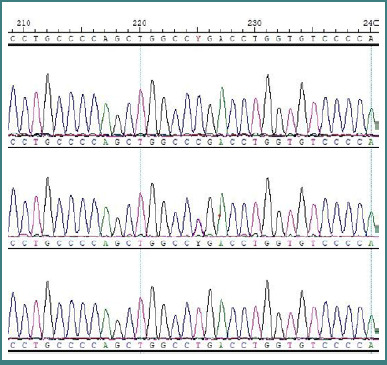
Sequencing results of partial sequence of the COL7A1 gene. Panel A shows a wild-type sequence of the *COL7A1* gene in healthy control individuals. Panel B is a c.409C>T in the *COL7A1* gene in a heterozygous state in parents of the affected individuals. A homozygous mutation (c.409C>T) in the affected individuals is presented in panel C.

The enrichment kit targeted the 44 Mb human genomic region and covered coding exons of >20,000 genes from the RefSeq and Consensus Coding Sequence (CCDS) databases. More than 80 million reads were generated. 96.8% of bases were covered with >10X coverage. By analyzing the WES data, a homozygous nonsense variant (c.409C>T) in exon 3 of the *COL7A1* gene was identified as a potential candidate variant based on previous reports of RDEB cases. The nonsense variant was predicted to introduce an immediate stop codon at position 137 (p.Arg137*). The variant was confirmed in a homozygous state in both patients and a heterozygous state in the parents and the unaffected sibling using Sanger sequencing (Figure 4).

## Discussion

Epidermolysis bullosa is a group of heterogeneous hereditary skin disorders of epithelial fragility [14,15]. Considerable variations in clinical presentation and the underlying genetic mutations across different forms of EB have been observed [16-19]. In this study, we examined individuals with a suspected autosomal recessive form of EB, and DNA analysis was conducted to identify the underlying mutation. Due to the lack of a skin biopsy and histological examination, we could not specifically determine the type of EB; therefore, WES was directly performed. Genetic analysis identified a potentially pathogenic homozygous nonsense mutation (c.409C>T) in the *COL7A1* gene and thus supported us in the conclusive diagnosis of recessive dystrophic EB (RDEB).

The homozygous variant (p.Arg137*) identified in this study has been reported earlier in a compound heterozygous state (p.Arg137*/p.Gln641*) in a male Japanese patient [20]. These protein-truncating compound heterozygous nonsense mutations (p.Arg137*/p.Gln641*) have been shown to cause a complete lack of collagen expression in the skin, likely due to nonsense-mediated mRNA decay [20]. If the mRNA escapes this decay mechanism, the resultant protein would be extremely short, comprising only 137 amino acids out of 2,944 and lacking all fibronectin-type domains.

Various mutations, including single nucleotide variants (mostly glycine substitution in the triple helix domain), deletions, or insertions, have been identified [21]. Mutations in *COL7A1* may result in premature termination codons, glycine substitutions, or splicing errors. Severe cases of RDEB are often caused by homozygous mutations leading to premature translational termination [22]. We have recently reported an apparent missense mutation in the *COL7A1* gene, which results in a splicing error and thus causes severe RDEB [23].

DEB can be inherited in autosomal recessive (RDEB) and autosomal dominant (DDEB) forms. Interestingly, both RDEB and DDEB are caused by mutations in the *COL7A1* gene encoding type VII collagen of the dermo-epidermal junction [24]. However, patients with RDEB show more severe clinical features, including recurrent and chronic open wounds. These are causing significant morbidity, impaired quality of life, and early mortality [25]. Mutations in *COL7A1* cause anchoring fibril defects and subsequent cutaneous sub-epidermal blisters. Healing may occur but with extensive scarring and milia formation. Repeated blisters result in chronic ulcers and joint contractures in severe cases [26]. Mitten hand and foot deformities are frequently observed in DEB [27]. Involvement of epidermal appendages (hairs and nails) is also commonly seen. Moreover, extra epidermal systems, including respiratory, urogenital, and digestive systems, have also been reported. Therefore, DEB is a multisystem disorder and thus is the most devastating type of EB [26].

## Conclusion

In this study, both affected siblings were diagnosed with a severe form of RDEB based on clinical symptoms combined with WES. This led to a nonsense mutation, identified for the first time in the Pakistani population, thus enlarging the mutation spectrum for RDEB in Pakistan. This study demonstrates that whole exome sequencing analysis is imperative in resolving clinically and genetically heterogeneous diseases.

## Personal thanks

We would like to thank all members of the family participating in this study and the Higher Education Commission (HEC).
