# Supplementary material for: A homozygous nonsense mutation identified in COL7A1 in a family with autosomal recessive dystrophic epidermolysis bullosa
Source: J Med Life. 2024 Sep;17(9):892–6. doi: 10.25122/jml-2024-0090 (PMC11611058; doi:10.25122/jml-2024-0090)
Supplement: Supplementary file 1 [file JMedLife-17-892-s001.pdf]

Table 1. Mutation details, demographic data, and clinical features of both patients

28 Patient	IV:1	IV:2
**Age (Years)**	10	12
**Sex**	Male	Female
**Geographic Origin**	Baluchistan, Pakistan	Baluchistan, Pakistan
**Variant**	(c.409C>T); (p.Arg137*)	(c.409C>T); (p.Arg137*)
**Variant type**	nonsense variant	nonsense variant
**Inheritance**	Autosomal recessive	Autosomal recessive
**Phenotypic Features**		
**Face**	Eczematous lesion, Conjunctivitis	Eczematous lesion, Conjunctivitis, Eyelid ulcerations
**Mouth**	Oral blisters	Oral blisters
**Hands**	Scaring, Digital fusion, Pseudosyndactyly, Mitten deformities, Amputation of 1st phalanx	Scaring, Digital fusion, Pseudosyndactyly, Mitten deformities, Amputation of 1st phalanx
**Feet**	Scaring, Digital fusion, Pseudosyndactyly, Mitten deformities, Auto-amputation of digits	Scaring, Digital fusion, Pseudosyndactyly, Mitten deformities, Auto-amputation of digits
**Skeletal**	Joint contractures	Joint contractures
**Skin**	Dystrophic epidermolysis bullish, recurrent Blistering, Erosions, Skin fragility, Severe Atrophic scarring, Milia, Mucosal lesions	Dystrophic epidermolysis bullish, recurrent Blistering, Erosions, Skin fragility, Severe Atrophic scarring, Milia, Mucosal lesions

© 2024 by the authors. This article is an open access article distributed under the terms and conditions of the Creative Commons Attribution (CC BY 4.0) license.
